# Prediction of all-cause mortality from 24 month trajectories in patient-reported psychological, clinical and quality of life outcomes in uveal melanoma patients

**DOI:** 10.1007/s10865-021-00252-8

**Published:** 2021-08-27

**Authors:** Stephen L. Brown, Peter L. Fisher, Laura Hope-Stone, Heinrich Heimann, Rumana Hussain, M. Gemma Cherry

**Affiliations:** 1 Department of Psychological Sciences, University of Liverpool, Liverpool, L69 3GB UK; 2 Liverpool Ocular Oncology Centre, Liverpool University Hospitals NHS Foundation Trust, Liverpool, Merseyside, L7 8XP UK

**Keywords:** Cancer, Mortality, Patient reported outcomes, Anxiety, Depression, Quality of life

## Abstract

A number of patient-reported outcomes (PROs) predict increased mortality after primary cancer treatment. Studies, though, are sometimes affected by methodological limitations. They often use control variables that poorly predict life expectancy, examine only one or two PROs thus not controlling potential confounding by unmeasured PROs, and observe PROs at only a single point in time. To predict all-cause mortality, this study used control variables affording good estimates of life expectancy, conducted multivariate analyses of multiple PROs to identify independent predictors, and monitored PROs two years after diagnosis. We recruited a consecutive sample of 824 patients with uveal melanoma between April 2008 and December 2014. PROs were variables shown to predict mortality in previous studies; anxiety, depression, visual and ocular symptoms, visual function impairment, worry about cancer recurrence, and physical, emotional, social and functional quality of life (QoL), measured 6, 12 and 24 months after diagnosis. We conducted Cox regression analyses with a census date of December 2018. Covariates were age, gender, marital and employment status, self-reported co-morbidities, tumor diameter and thickness, treatment modality and chromosome 3 mutation status, the latter a genetic mutation strongly associated with mortality. Single predictor analyses (with covariates), showed 6-month depression and poorer functional QoL predicting mortality, as did 6–12 month increases in anxiety and 6–12 month decreases in physical and functional QoL. Multivariate analyses using all PROs showed independent prediction by 6-month depression and decreasing QoL over 6–12 months and 12–24 months. Elevated depression scores six months post-diagnosis constituted an increased mortality risk. Early intervention for depressive symptoms may reduce mortality.

**Supplementary Information:**

The online version contains supplementary material available at 10.1007/s10865-021-00252-8.

## Introduction

Patient reported outcomes (PROs) are subjective measures of physical and psychological health. A range of PROs assessed either after diagnosis or during or after primary cancer treatment have been shown to predict patient mortality independently of demographic and clinical prognostic variables (Efficace, et al., 2021; Nakaya, 2014).^1^ Systematic reviews show that higher self-reported depression and anxiety predict increased mortality across a range of cancers (Pinquart & Duberstein, 2010a; Wang, et al., 2020). Primary studies have found that self-reported physical symptoms (McFarland et al., 2020), fear of cancer recurrence (Kim, et al., 2020), and poorer quality of life (QoL) (Groenvold, et al., 2007; Quinten, et al., 2009; Ratjen, et al., 2018) also predict mortality.

Two proposed mechanisms link PROs to mortality. First, poorer PROs may be associated with higher psychological stress, which contributes to chronically heightened physiological stress responses causing hypothalamic–pituitary–adrenal (HPA) axis dysregulation and corticosteroid overproduction (Pinquart & Duberstein, 2010a). These lead to mortality through immune suppression and vascular and organ damage (Spiegel & Giese-Davis, 2003). The second mechanism is unhealthy behavior. Poorer PROs have been linked to poorer compliance with clinical advice, such as medication use and screening attendance, and poorer health behaviors such as unhealthy eating (Strine, et al., 2008).

Previous studies linking PROs to mortality are potentially subject to confounds. First, PROs can co-vary with biomedical attributes, such as cancer progression, which are often difficult to measure but may nonetheless represent the true causes of mortality (Pinquart & Duberstein, 2010a). Most cancer mortality studies control for the effects of cancer site, stage and treatment, but these variables are often only poor or moderate prognostic indicators (Hui, 2015). More stringent hypothesis testing requires control variables that are stronger prognostic indicators. A second potential confound arises because most previous studies predict mortality from only one or two PROs. Many PROs, such as anxiety, depression, experience of symptoms, fear of cancer and QoL, appear to be strongly correlated (Brown, Fisher, Hope-Stone, Hussain, Heimann, et al., 2020). Studies that assess multiple predictors often show that good univariate predictors are not multivariate predictors because they share predictive variance with other PROs (Sehlen, et al., 2012; Walker, et al., 2020). Thus, existing studies linking PROs to mortality may be confounded by other, unmeasured, PROs, and it is unclear which PROs are most likely to contribute to mortality. A more precise understanding of relationships between PROs and mortality can be achieved by identifying PROs that predict mortality independently of others, thus strengthening (although not proving) causal claims. Thus, it is important to examine multiple PROs simultaneously using multivariate techniques, with appropriate prognostic controls, to identify independent predictors.

Third, many studies measure PROs at a single timepoint, usually at diagnosis or during or after primary treatments such as surgery. However, PROs change over time (Brown, et al., 2020), and it is unclear whether a single timepoint can entirely represent PROs over the full survivorship period. Anxiety and depression, for example, can be elevated immediately after diagnosis but reduce within three to six months (Beesley, et al., 2020; Crane, et al., 2019), whilst post-treatment physical symptoms and functional impairments can worsen (Brown, et al., 2020). Documenting PROs at multiple timepoints can provide a more trustworthy representation of patients’ experiences in survivorship. A second advantage of multiple timepoints is the ability to assess impacts of temporal changes in individuals’ PROs. Repeated observation studies show temporal variability in associations with mortality (Eldridge, et al., 2019; Raffel, et al., 2017; Zhu, et al., 2020), suggesting that the timeframes of elevations in PROs might influence mortality. This would have implications for the timing of interventions.

## Current study

We aimed to examine associations between mortality and PROs, similar to those used in previous studies, whilst addressing the above methodological limitations. We used uveal melanoma (UM) patients because strong prognostic indicators are available. UM has about a 50% ten-year mortality rate, mostly attributable to metastatic disease which occurs most commonly in the liver (Kujala et al., 2003). Risk of metastatic disease is principally determined by tumor size and a single chromosomal mutation, monosomy 3, which itself is strongly associated with tumor size (Damato et al., 2010). Using M3 status and tumor characteristics, prognostication of life expectancy is sufficiently accurate to provide reliable individual estimates (DeParis, et al., 2016). We added M3 status and tumor diameter and thickness to control variables similar to those used in previous studies.

PROs in this study were anxiety and depression, visual and ocular symptoms, functional limitations, worry about recurrence (WREC) and physical, emotional, social and functional quality of life (QoL). We examined univariate relationships, predicting mortality from each PRO separately, to assess whether these predicted mortality independently of control variables only. We then used multivariate analyses, using all PROs as predictors, to assess which PROs predicted mortality independently of both control variables and other PROs. Instead of a single time point, we measured participants’ PROs over two years, at 6, 12 and 24 months after diagnosis. If worsening of individuals’ PRO scores between two timepoints, relative to the remainder of the sample, predicts mortality (controlling PRO scores at the first timepoint) this would indicate that mortality might be influenced by these changes.^2^

Poorer PROs are indicated by higher scores for anxiety, depression, visual/ocular symptoms, visual function limitations, worry about cancer recurrence, and by lower QoL scores. We hypothesized that higher mortality would be independently predicted by poorer initial PRO scores six months after diagnosis. We predicted higher mortality in participants whose scores deteriorated, relative to the remainder of the sample, between 6–12 and 12–24 months.

## Method

Data were obtained from an audit of PROs, approved by the Liverpool Central Ethics Committee (03/06/072/A). Consecutive eligible patients were posted reply-paid questionnaires 6, 12 and 24 months after diagnosis. Inclusion criteria were: adult patients, aged 18 years and over, resident in England or Wales, treated for posterior (choroid or ciliary body) UM at the Liverpool Ocular Oncology Centre (LOOC) between April 1st 2008 and December 31st 2014. Treatment was based on the protocol described in Damato and Heimann (2013). Ruthenium plaque radiotherapy or proton beam radiotherapy were first considered. If the tumor was unsuitable for radiotherapy, patients underwent trans-scleral local resection, trans-retinal endoresection or enucleation (eye removal). Prognostic testing to determine M3 status was offered and explained to patients, who chose whether to undergo prognostication. Prognostic outcomes were provided before the six-month observation, and explained to patients by LOOC staff or their clinical oncology team. Patients with a poor prognosis were given the option to attend a regular liver screening program.

## Measures

### Demographic, clinical and treatment variables

Age, gender, relationship status, employment status, and primary treatment type were recorded at diagnosis. Patients were asked if they had health problems (co-morbidities) unrelated to their eye. Largest basal tumor diameter and tumor thickness were measured in millimeters. Chromosomal mutation M3 is deletion of one of the normal two maternal or paternal copies of chromosome 3; disomy 3 (D3) is the normal two chromosomes. M3 ten-year disease specific mortality is over 50% (if tumor diameter is > 12 mm), whilst the D3 mortality rate is normal. At the time of this study, only liver resection could prolong life for metastatic uveal melanoma patients, and only for a small minority. Outcomes of prognostic testing were M3, D3 or unknown, the latter comprising patients who did not wish to be tested, when tumor characteristics prohibited prognostication or whose cytogenetic test failed.

### Anxiety and depression

Anxiety and depression were measured using subscales of the Hospital Anxiety and Depression Scale (HADS) (Zigmond & Snaith, 1983). Each has seven items scored from 0 to 3 with higher scores signifying greater symptomology (range = 0–21). Both subscales predict diagnosed cases with good sensitivity and specificity, with clinical cut-off scores of eight and above (Vodermaier & Millman, 2011).

Post-treatment visual and ocular symptoms, visual functional limitations and worry about recurrence were measured using sub-scales of the European Organisation for Research and Treatment for Cancer Ophthalmic Quality of Life^3^ questionnaire (EORTC QLQ-OPT 30; Brandberg et al., 2004), designed for UM patients and validated in UM samples (Chmielowska et al., 2013).

### Symptoms

Subscales used in this study were: ocular irritation (6 items, e.g., ‘Discharge from the treated eye’, Cronbach alphas; 6 months 0.70, 12 months 0.73, 24 months 0.77); visual impairment (4 items e.g., ‘Were you troubled by any defects in side vision’, Alphas; 0.75, 0.73, 0.72); and headache (single item ‘Did you have headaches?’). To simplify analysis, we used confirmatory factor analysis to test a single latent factor model consisting of the subscales. A final single factor model with fixed error covariances for the above sub-scales showed satisfactory fit, *Χ*^2^_(2.65)_ = 15.87, CFI = 0.98, RMSEA = 0.06. For the analyses, we computed a single mean of the three subscales that we labelled visual and ocular symptoms.

### Functional impairment

The EORTC QLQ-OPT 30 sub-scale measuring functional limitations was also used (5 items, e.g., ‘Difficulty seeing steps or pavements?’ Alphas; 0.93, 0.92, 0.92).

### Worry about cancer recurrence (WREC)

We used the EORTC QLQ-OPT 30 4-item sub-scale measuring worry about local and secondary cancer recurrence (WREC); Alphas; 0.86, 0.82, 0.82). An item on concern about loss of the eye was excluded because it was not relevant to enucleated patients. The remaining three items were used: ‘Were you worried about your health in the future?’; ‘Were you worried about the tumor recurring in the treated eye?’ and ‘Were you worried about the tumor recurring in other areas of your body?’ Response format for all EORTC QLQ-OPT 30 items is ‘Not at all’, ‘A little’, ‘Quite a bit’ and ‘Very much’, scored 1–4, respectively, and all items were worded in terms of poorer outcomes.

### QoL

QoL was assessed using the physical, social/family, emotional and functional well-being subscales of the Functional Assessment of Cancer Therapy –General (FACT-G) Scale (Cella, et al., 1993; Webster & Yost, 2003). 27 items are scored on a 5-point Likert scale, indicating general health, anchored by the terms ‘not at all’ and ‘very much’. Subscale items include physical well-being (7 items, e.g., ‘I am forced to spend time in bed’, Alphas; 0.79, 0.82, 0.83); Social/family well-being (7 items, e.g., ‘I get support from my friends’, Alphas; 0.82, 0.86, 0.87); emotional well-being (6 items, e.g., ‘I am losing hope in the fight against illness); and functional well-being (7 items, e.g. ‘I am able to enjoy life’). Higher scores indicate greater QoL. All subscales have been extensively used in cancer populations and show good reliability and validity (Cella, et al., 1993; Webster & Yost, 2003).

All-cause mortality was ascertained from information provided by the England and Wales death registry.

## Analysis

As data were sometimes skewed (maximum skew = 2.37), we used linear mixed modeling to examine temporal trends in predictor variables, treating 6, 12 and 24 month observations as random observations. Linear mixed models are robust against skews of at least 3 (Schielzeth, Dingemanse, Nakagowa, et al., 2020). Cox regression was used to estimate the adjusted daily hazard ratio of predictor variables (differential likelihood of death per day attributable to the predictor variable). SPSS version 27 was used for all procedures. The census period for each participant was from the 24-month timepoint to a census date of 31 December 2018. Initial covariates were age, sex, marital status, employment status, tumor diameter and thickness, treatment modality and chromosome 3 status (D3, M3, unknown), with those not contributing to the overall chi-square or -2log likelihood statistic discarded for the final analyses. Predictors were 6, 12 and 24-month scores on all predictor variables. We used single variable and multivariate analyses. Single variable analyses establish whether individual PROs at the 6, 12, and 24 month steps predict mortality independently of the covariates, without including other PROs in the model. Multivariate analyses include all PROs, and identify prediction independently of the covariates and other PROs used in the study.

In single variable and multivariate analyses, variables were entered hierarchically in four steps: covariates were entered at the first step (these were common to all analyses), 6 month predictors at the second step, 12 month predictors at the third step and 24 month predictors at the fourth. Hierarchical entry allows statistical control of variables entered at previous and current steps. Univariate analyses were conducted by entering covariates then single PROs at each step (6, 12 and 24 months). Multivariate analyses were performed by adding all predictors at each step. Results are displayed using adjusted hazard ratios and 95% confidence intervals. Parameters are presented as they appeared at the step on which they were entered, not the final model.

We included all patients if they provided data at the 6-month timepoint. Replacement values were estimated for 52 participants who missed the 12 month time point, 93 who missed the 24 month time point and 131 who missed both timepoints. Additionally, 30 patients were excluded who completed 6 and 12-month timepoints but died before the 24 month timepoint. Replacement was by fully conditional specification multiple imputation using linear regression for continuously distributed variables. Ten imputations (corresponding to approximately 10% missing data) were based on age, gender, tumor diameter and thickness, all predictor variables at all timepoints, outcome (survival or death) and the baseline hazard function (White & Royston, 2009), with pooled data used in analyses.

## Results

Table 1 shows demographic and clinical characteristics of the 824 patients included in the analyses. Mean age was 68.94 (*SD* = 12.39) with approximately equal representation of males and females. 225 (32.5%) of participants had a M3 melanoma. 224 patients (27.18%) died before the end of census, of whom 126 were M3. Mean number of days until the end of census for survivors was 2721.87 (SD = 794.34). Mean number of days until death was 1377.54 (SD = 739.42).

**Table 1:** Demographic and Clinical Characteristics of Participants

	Number	Percentage
*Sex*		
Males	435	52.7%
Females	389	47.2%
*Marital status*		
Married or with partner	595	72.1%
Widowed	110	13.3%
Divorced/separated	85	13.0%
Single	2	0.3%
*Treatment*		
Enucleation	177	25.5%
Plaque radiotherapy	304	43.9%
Proton beam radiotherapy	155	22.4%
Resection	35	5.1%
Other	22	3.2%
*Chromosome 3 status*		
Monosomy 3	225	33.5
Disomy 3	178	25.7
Unknown	290	41.8
*Eye*		
Left	401	48.7
Right	423	51.3
Tumor diameter (mm)	Mean = 11.85	*SD* = 3.89
Tumor thickness (mm)	Mean = 4.46	*SD* = 3.37

Table 2 shows 6, 12 and 24 month means and *SD*s for predictor variables. Population norms are available for anxiety, depression and QoL scales. Anxiety and depression mean scores on the HADS were similar to Crawford et al’s (2001) UK (non-clinical) population norms of 6.14 for anxiety and 3.68 for depression (*n* = 1,792). QoL norms are not available for the UK, but means were similar to roughly comparable Australian general population norms (Janda et al., 2009). Visual and ocular symptoms and WREC scores declined slightly over two years. Emotional QoL scores increased. Appendix 1 shows correlations between predictors.

**Table 2:** Means, SDs and Linear Trends of Predictor variables

	6 Month	12 Month	24 Month	*F*(2,1459)^$^
Anxiety	5.48 (4.24)	5.17 (4.14)	5.16 (3.96)	1.45, *p* = .234
Depression	3.46 (3.47)	3.30 (3.43)	3.28 (3.35)	0.62, *p* = .536
Anxiety % clinical concern*	29.0	24.2	24.6	
Depression % clinical concern	13.8	11.8	12.5	
Visual and ocular symptoms	1.59 (0.50)	1.53 (0.42)	1.50 (0.42)	7.03, *p* < .01
Visual function limitations	1.66 (0.74)	1.62 (0.67)	1.60 (0.61)	1.27, *p* = .279
Worry cancer recurrence	2.44 (.92)	2.21 (0.80)	2.11 (0.74)	30.58, *p* < .01
FACT G physical	24.88 (3.89)	24.96 (4.13)	25.20 (3.74)	1.43, *p* = .239
FACT G social	23.17 (5.57)	23.04 (5.73)	23.02 (5.26)	0.25, *p* = .861
FACT G emotional	18.40 (4.60)	18.99 (4.30)	19.25 (3.88)	7.82, *p* < .01
FACT G functional	21.17 (6.23)	21.55 (6.23)	21.84 (5.23)	2.60, *p* = .074

^*^Patients with scores of 8 or greater on HADS anxiety and depression indicating clinical concern (Vodermaier & Millman, 2011). ^$^*F* statistic derived from linear mixed modeling

## Predictors of mortality

### Control variables

Step 1, consisted of demographic and clinical control variables. Variables not used, because their inclusion did not contribute to the overall chi-square or -2log likelihood statistic, were sex, marital status, employment status, treatment, tumor thickness and self-reported other health problems. The step showed multivariate prediction of mortality; Chi-sq (9*df*) of 247.79, and -2log likelihood of 2,587.84. This step was used in all single and multiple predictor analyses. Greater proportional hazard was associated with increasing age (adjusted hazard ratio = 1.03, 95% CI = 1.01, 1.04) and tumor diameter (HR_(adj)_ = 1.21, 95% CI = 1.16, 1.27), but not sex (HR_(adj)_ = 0.86, 95% CI = 0.66, 1.23). Chromosome 3 status predicted mortality. Compared to the no prognosis group, lower mortality was associated with D3 (HR_(adj)_ = 0.22, 95% CI = 0.12, 0.39) but M3 participants did not show greater mortality (HR_(adj)_ = 1.29, 95% CI = 0.90, 2.13). Compared to enucleation, lower hazard was associated with being treated with proton beam radiotherapy (HR_(adj)_ = 0.45, 95% CI = 0.21, 0.94) and resection (HR_(adj)_ = 0.30, 95% CI = 0.13, 0.67). Plaque radiotherapy (HR_(adj)_ = 0.0.59, 95% CI = 0.27, 1.31) and other treatment (HR_(adj)_ = 0.76, 95% CI = 0.30, 1.92) did not predict mortality.

Steps 2, 3 and 4 consisted of 6, 12 and 24 month predictors respectively. Table 3 shows hazard ratios and 95% confidence intervals of step 2–4 predictors in the univariate and multivariate survival analysis.

**Table 3:** Univariate and Multivariate Hazard Ratios and 95% Confidence Intervals (smaller font) in Survival Analyses

	Single variable analyses^†^		
	6 Mths. (Step 2)	12 Mths. (Step 3)	24 Mths. (Step 4)
Anxiety	0.96 1.00 1.03	1.021 1.08* 1.14	0.93 0.99 1.06
Depression	1.02 1.06* 1.11	0.99 1.05 1.12	0.96 1.031 1.11
Visual & ocular symptoms	0.891 1.24 1.72	0.83 1.36 2.22	0.47 0.816 1.38
Visual function limitations	0.82 1.01 1.24	1.02 1.36* 1.83	0.53 0.74 1.04

	Multivariate analyses^†^		
	6 Mths. (Step 2)	12 Mths. (Step 3)	24 Mths. (Step 4)
Anxiety	0.86 0.90* 0.96	0.93 1.01 1.09	0.92 0.98 1.06
Depression	1.01 1.09* 1.17	0.85 0.93 1.01	0.92 0.98 1.06
Visual & ocular symptoms	0.63 0.98 1.51	0.44 079 1.41	0.42 0.99 1.69
Visual function limitations	0.86 1.10 1.40	0.80 1.13 1.60	0.55 0.88 1.16
Worry cancer recurrence	0.77 0.92 1.13	0.65 0.86 1.13	0.75 1.02 1.38
FACT G physical	0.95 0.98 1.05	0.88 0.93* 0.98	0.89 0.94* 0.98
FACT G social	0.95 0.97 1.00	0.99 1.03 1.07	0.97 1.01 1.04
FACT G emotional	0.92 0.96 1.01	0.89 0.95 1.01	0.97 1.03 1.10
FACT G functional	0.97 1.01 1.04	0.93 0.97 1.01	0.93 0.96 1.01

Full parameters for all models are available in Appendix 2. Parameters are presented as they appeared after the step, not in the final model; Step 1 control variables were identical for each analysis (see text for parameters); ^†^Single variable analyses are measures of a single predictor at 6, 12 and 24 month steps, multivariate is the entry of all predictors at each step; * *p* < .05

### Single variable analyses

Greater mortality was associated with higher depression scores (HR_(adj)_ = 1.06) and lower physical QoL (HR_(adj)_ = 0.96) and functional QoL (HR_(adj)_ = 0.97) at six months, 12 month anxiety (HR_(adj)_ = 1.08) and visual function limitation scores (HR_(adj)_ = 1.36), and lower 12 month physical (HR_(adj)_ = 0.92) and functional QoL (HR_(adj)_ = 0.968). Visual and ocular symptoms and worries about recurrence did not predict mortality.

### Multivariate analyses

Significant multivariate increases in prediction occurred at 6 (Chi-sq change (7 *df*) = 27.13, *p* < 0.01), and 12 month steps (Chi-sq change (7 *df*) = 23.61, *p* < 0.01) but not the 24 month step (Chi-sq change (7 *df*) = 12.96, *p* = 0.105). Of the predictor variables, six-month depression (HR_(adj)_ = 1.09) predicted greater mortality, as lower physical QoL at 12 (HR_(adj)_ = 0.93) and 24 months (HR_(adj)_ = 0.94). Lower anxiety at 6 months inversely predicted mortality, but as the univariate analysis did not show prediction, this is probably attributable to a multivariate suppression effect.^4^ Neither visual and ocular symptoms, visual function limitations nor worry about cancer recurrence predicted mortality.

## Discussion

We examined PROs as predictors of mortality using three methodological improvements over previous studies; stronger prognostic control variables, multivariate analyses to identify independent predictors and longitudinal assessment of predictors. All variables used have predicted mortality in previous studies (Nakaya, 2014; McFarland, et al., 2020; Kim, et al., 2014; Efficace, et al., 2021; Wang, et al., 2020) but few did so in this study, possibly due to our enhanced statistical controls. The univariate analysis showed that greater anxiety, depression, visual function limitations and poorer functional and physical QoL predicted mortality. The multivariate analysis showed that depression and physical QoL independently predicted mortality. Longitudinal analyses showed the importance of events that occurred during survivorship. Mortality was predicted by 12 and 24 month physical QoL (multivariate), and 6–12 month increases in anxiety (univariate only) and decreases in functional QoL (univariate only), suggesting that reductions in QoL and increases in anxiety during the study predicted mortality.

As mentioned, a key finding is that most PROs did not predict mortality after our stringent protections against confounding. Neither visual and ocular symptoms, worry about cancer recurrence, nor social or emotional QoL predicted mortality. We emphasize that this finding does not necessarily constitute a failure to replicate previous studies, because we did not use exactly the same PRO measures or the same populations. Thus, we cannot definitively claim that previous findings would not hypothetically survive the enhanced statistical controls we used. Nonetheless, in the context of our findings, these variables are not yet established to be good predictors of mortality.

An exception is our finding that initial depression scores predicted mortality. This is commonly found in the literature (Pinquart & Duberstein, 2010a; Wang, et al., 2020), and our additional control variables lend confidence that the effect is probably not attributable to confounding. It is notable that changes in depression after the six-month time point did not influence mortality. This suggests that mortality might be influenced by depressive symptoms manifest either early in cancer survivorship or before diagnosis, and that subsequent reductions in these are not associated with lower mortality.

Consistent with some previous studies (Groenvold, et al., 2007; Ratjen, et al., 2018), higher QoL may be protective against mortality in cancer patients. Six-month QoL scores generally were not associated with mortality (with the exception of functional well-being in the univariate analysis). However, relative reductions in physical QoL between 6 and 12 months predicted higher mortality in the multivariate analysis and functional QoL between 6 and 12 months in the univariate analysis. Social and emotional QoL did not predict mortality. It is notable that QoL domains pertaining to physical health and functioning predicted mortality, but social and emotional domains did not. It is possible that poorer perceptions of health and functioning may represent psychological influences on mortality; whereby physical and functional QoL represent physically-felt manifestations of unknown psychological origins. However, it is also possible that poorer physical QoL reflects poorer physical health that preceded either metastasis or an unrelated disease that contributed to death. If the latter is true, PROs may not cause physical disease, but be useful screening tools to provide early indications of it.

Six to twelve-month increases in anxiety and visual function limitations and 6 month functional QoL were univariate but not multivariate predictors. This finding is similar to some previous research. Anxiety, for example, has previously been associated with mortality (Wang, et al., 2020) but does not predict mortality independently of depression (see also Walker, et al., 2020). In sharing predictive variance with other PROs these variables may possibly have a causal role, but evidence is not as strong as for independent predictors.

We did not show univariate links between mortality and our participants’ concerns about ocular symptoms and worry about recurrence. This is possibly because our control variables enabled a stricter hypothesis test (Kim et al. and McFarland et al. used disease subtype, site, stage and treatment). These variables may not be good predictors of mortality. We concede that our findings could be specific to UM, where illness and treatment effects are not as severe as other cancers. Patients do not undergo prolonged chemotherapy or radiotherapy and symptoms and functional problems are constrained to the eye and sight. These PROs may predict mortality in cancers where illness and treatment effects are more severe. WREC may not be predictive in our study because we controlled for M3 status, an objective measure of recurrence risk that is known to participants.

## Limitations

Although we took precautions against confounding, we cannot fully eliminate the possibility that unmeasured biomedical variables, associated with either UM or unrelated illnesses, explain our findings. We did not examine social support, a known predictor of mortality (Pinquart & Duberstein, 2010b; Uchino, 2006), because it is not a disease or treatment outcome. Nonetheless social support might moderate our findings by reducing negative impacts of depression. Our linkage to the England and Wales death records limits ascertainment of death to residents only, and deaths outside this jurisdiction were not recorded.

## Research and clinical implications

The most widely cited mechanism for links between PROs and mortality is the deleterious influence of physiological stress, caused by PROs such as depression, on HPA and immune function (Pinquart & Duberstein, 2010a), thus accelerating metastatic spread. Depression and poorer QoL are associated with HPA and immune dysregulation (Spiegel & Giese-Davis, 2003; Wang, et al., 2020). As with most previous studies, we cannot eliminate the possibility that behavioral risk factors, such as non-compliance with medications or substance use, linked to depression might be the proximal causes of mortality. However, mediation by neither physiological stress nor health behavior has not been formally tested in the literature. To form stronger conclusions about mechanisms, future research, should examine mediational hypotheses regarding physiological stress and health behavior.

We can point to two clinical insights. Firstly, the timing of effects appears to be important. If depression does influence mortality, our findings suggest that this effect would probably be established by the first six months of survivorship. Depression reductions after this point were not associated with improved mortality, suggesting that any interventions should be delivered early in survivorship. Conversely, QoL changes after six months were predictive, thus identifying the reasons for relative decline in QoL, and possibly anxiety, after six months could be important for intervention. If declines in physical QoL represent somatization of psychological distress leading to mortality, distress could potentially be identified and treated. If it provides early indication of disease progression, there may be diagnostic implications.

Second, overall depression and anxiety scores were comparable to community norms (Crawford, et al., 2001). This suggests that variations in anxiety and depression within normal population parameters are potentially important in predicting mortality. Psychological services for cancer patients have traditionally focused on clinical and sub-clinical groups, although there is some evidence in cancer generally (Bonacchi, et al., 2010) and UM in particular that psychological need is present in patients who do not meet clinical thresholds (Hope-Stone et al., 2019). Our findings suggest that recent developments in widening population access through accessible but effective interventions, such as online psychological self-help programmes (White, et al., 2020), may be helpful.

Overall, this study provided only limited support for the proposal that PROs are associated with mortality, with only depression and physical QoL being significant multivariate predictors. Causality could be established by research, that elucidates mediation of this effect, and shows that reducing depression and increasing physical QoL can improve mortality. Such research could underpin interventions that might improve mortality.

## Supplementary Information

Below is the link to the electronic supplementary material.

Supplementary file1 (DOCX 55 kb)

## Footnotes

1 These studies employ Cox regression or similar models that predict the risk of mortality per unit of time. It is important to note that PRO may delay rather than prevent death.

2 Change is indicated by significant 12 or 24-month variables. Relative worsening or improvement does not necessarily connate change in absolute values, as, for example, an individual may show relative deterioration even if their scores remain stable if the rest of the sample improves at a greater rate.

3 Previous studies use the term QoL for illness-specific measures of symptomology such as the EORTC and generic scale such as the FACT-G. We use the term QoL to refer to the generic FACT-G sub-scales, not the EORTC.

4 Anxiety and depression scores were correlated (*r* between .62 and .65). Conducting the analysis without anxiety, depression remained a predictor. Conducting the analysis without depression scores led to a positive but non-significant relationship between anxiety and mortality, supporting a suppressor effect interpretation.
