# Supplementary material for: Prediction of all-cause mortality from 24 month trajectories in patient-reported psychological, clinical and quality of life outcomes in uveal melanoma patients
Source: J Behav Med. 2021 Aug 27;45(1):115–23. doi: 10.1007/s10865-021-00252-8 (PMC8818627; doi:10.1007/s10865-021-00252-8)
Supplement: Supplementary file 1 — Supplementary file1 (DOCX 55 kb) [file 10865_2021_252_MOESM1_ESM.docx]

**APPENDIX 1 - INTERCORRELATIONS AMONGST PREDICTOR VARIABLES**

|  | 2. | 3. | 4. | 5. | 6. | 7. | 8. | 9. | 10. | 11. | 12. | 13. | 14. | 15. | 16. | 17. | 18. | 19. | 20. | 21. | 22. | 23. | 24. | 25. | 26. | 27. |
| --- | --- | --- | --- | --- | --- | --- | --- | --- | --- | --- | --- | --- | --- | --- | --- | --- | --- | --- | --- | --- | --- | --- | --- | --- | --- | --- |
| 6 Month |  |  |  |  |  |  |  |  |  |  |  |  |  |  |  |  |  |  |  |  |  |  |  |  |  |  |
| 1. Anxiety | .65 | .46 | .33 | .53 | -.52 | -.23 | -.71 | -.52 | .78 | .49 | .40 | .25 | .44 | -.38 | -.20 | -.55 | -.39 | .73 | .46 | .39 | .25 | .45 | -.33 | -.18 | -.52 | -.32 |
| 2. Depression |  | .54 | .44 | .35 | -.68 | -.37 | -.56 | -.74 | .57 | .76 | .51 | .41 | .35 | -.55 | -.28 | -.48 | -.59 | .56 | .75 | .43 | .34 | .30 | -.48 | -.28 | -.44 | -.53 |
| 3. Symptoms |  |  | .58 | .44 | -.59 | -.19 | -.50 | -.46 | .45 | .45 | .73 | .51 | .40 | -.48 | -.17 | -.44 | -.37 | .47 | .44 | .66 | .48 | .36 | -.43 | -.13 | -.41 | -.32 |
| 4. Vis. Funct. |  |  |  | .30 | -.43 | -.11 | -.34 | -.39 | .34 | .39 | .50 | .75 | .34 | -.36 | -.06 | -.35 | -.31 | .33 | .39 | .45 | .65 | .22 | -.34 | -.04 | -.33 | -.27 |
| 5. WREC |  |  |  |  | -.35 | -.06 | -.65 | -.27 | .43 | .30 | .35 | .25 | .60 | -.30 | -.07 | -.45 | -.25 | .39 | .24 | .33 | .24 | .59 | -.21 | -.01 | -.45 | -.18 |
| 6. QoL Phys. |  |  |  |  |  | .26 | .50 | .60 | -.47 | -.58 | -.51 | -.37 | -.29 | .62 | .19 | .40 | .47 | -.49 | -.59 | -.48 | -.33 | -.29 | .60 | .21 | .43 | .44 |
| 7. QoL Soc. |  |  |  |  |  |  | .21 | .44 | -.21 | -.29 | -.17 | -.07 | -.05 | .19 | .58 | .17 | .31 | -.22 | -.32 | -.11 | -.04 | -.06 | .17 | .50 | .15 | .30 |
| 8. QoL Emot. |  |  |  |  |  |  |  | .48 | -.58 | -.44 | -.39 | -.25 | -.49 | .33 | .16 | .59 | .36 | -.53 | -.38 | -.33 | -.22 | -.48 | .27 | .14 | .56 | .28 |
| 9. QoL Funct. |  |  |  |  |  |  |  |  | -.45 | -.60 | -.45 | -.35 | -.28 | .44 | .34 | .39 | .64 | -.45 | -.58 | -.38 | -.28 | -.29 | .39 | .28 | .38 | .55 |
| 12 Month |  |  |  |  |  |  |  |  |  |  |  |  |  |  |  |  |  |  |  |  |  |  |  |  |  |  |
| 10. Anxiety |  |  |  |  |  |  |  |  |  | .62 | .46 | .34 | .47 | -.48 | -.25 | -.68 | -.49 | .79 | .50 | .41 | .32 | .44 | -.42 | -.19 | -.55 | -.40 |
| 11. Depression |  |  |  |  |  |  |  |  |  |  | .50 | .43 | .37 | -.69 | -.34 | -.59 | -.73 | .56 | .76 | .41 | .37 | .30 | -.53 | -.30 | -.45 | -.59 |
| 12. Symptoms |  |  |  |  |  |  |  |  |  |  |  | .63 | .45 | -.58 | -.20 | -.48 | -.48 | .41 | .42 | .66 | .49 | .33 | -.42 | -.16 | -.32 | -.32 |
| 13. Vis. Funct. |  |  |  |  |  |  |  |  |  |  |  |  | .40 | -.43 | -.07 | -.40 | -.40 | .28 | .38 | .45 | .71 | .24 | -.34 | -.03 | -.26 | -.28 |
| 14. WREC |  |  |  |  |  |  |  |  |  |  |  |  |  | -.40 | -.07 | -.62 | -.35 | .38 | .27 | .34 | .30 | .59 | -.23 | -.03 | -.42 | -.21 |
| 15. QoL Phys. |  |  |  |  |  |  |  |  |  |  |  |  |  |  | .25 | .56 | .62 | -.42 | -.55 | -.44 | -.33 | -.26 | .54 | .20 | .35 | .39 |
| 16. QoL Soc. |  |  |  |  |  |  |  |  |  |  |  |  |  |  |  | .26 | .45 | -.23 | -.31 | -.14 | -.06 | -.08 | .22 | .56 | .17 | .33 |
| 17. QoL Emot. |  |  |  |  |  |  |  |  |  |  |  |  |  |  |  |  | .52 | -.55 | -.47 | -.38 | -.31 | -.46 | .37 | .14 | .55 | .33 |
| 18. QoL Func. |  |  |  |  |  |  |  |  |  |  |  |  |  |  |  |  |  | -.42 | -.58 | -.35 | -.28 | -.26 | .42 | .32 | .34 | .59 |
| 24 Month |  |  |  |  |  |  |  |  |  |  |  |  |  |  |  |  |  |  |  |  |  |  |  |  |  |  |
| 19. Anxiety |  |  |  |  |  |  |  |  |  |  |  |  |  |  |  |  |  |  | .65 | .48 | .35 | .48 | -.49 | -.27 | -.66 | -.51 |
| 20. Depression |  |  |  |  |  |  |  |  |  |  |  |  |  |  |  |  |  |  |  | .46 | .41 | .28 | -.60 | -.38 | -.52 | -.69 |
| 21. Symptoms |  |  |  |  |  |  |  |  |  |  |  |  |  |  |  |  |  |  |  |  | .65 | .47 | -.56 | -.18 | -.49 | -.39 |
| 22. Vis. Funct. |  |  |  |  |  |  |  |  |  |  |  |  |  |  |  |  |  |  |  |  |  | .35 | -.46 | -.06 | -.40 | -.34 |
| 23. WREC |  |  |  |  |  |  |  |  |  |  |  |  |  |  |  |  |  |  |  |  |  |  | -.35 | -.05 | -.63 | -.29 |
| 24. QoL Phys. |  |  |  |  |  |  |  |  |  |  |  |  |  |  |  |  |  |  |  |  |  |  |  | .38 | .56 | .57 |
| 25. QoL Soc. |  |  |  |  |  |  |  |  |  |  |  |  |  |  |  |  |  |  |  |  |  |  |  |  | .23 | .48 |
| 26. QoL Emot. |  |  |  |  |  |  |  |  |  |  |  |  |  |  |  |  |  |  |  |  |  |  |  |  |  | .49 |

**APPENDIX 2 - FULL OUTCOMES OF THE MULTIVARIATE SURVIVAL ANALYSIS**

**ENTRY OF CONTROL VARIABLES**

| **Omnibus Tests of Model Coefficients^a^** | | | | | | | | | |
| --- | --- | --- | --- | --- | --- | --- | --- | --- | --- |
| -2 Log Likelihood | Overall (score) | | | Change From Previous Step | | | Change From Previous Block | | |
|  | Chi-square | df | Sig. | Chi-square | df | Sig. | Chi-square | df | Sig. |
| 2587.838 | 250.464 | 8 | .000 | 247.790 | 8 | .000 | 247.790 | 8 | .000 |
|  | | | | | | | | | |

| **Variables in the Equation** | | | | | | | | |
| --- | --- | --- | --- | --- | --- | --- | --- | --- |
|  | B | SE | Wald | df | Sig. | Exp(B) | 95.0% CI for Exp(B) | |
|  |  |  |  |  |  |  | Lower | Upper |
| Age | .025 | .006 | 17.053 | 1 | .000 | 1.026 | 1.013 | 1.038 |
| Tumor diameter | .190 | .022 | 74.568 | 1 | .000 | 1.209 | 1.158 | 1.262 |
| Treatment |  |  | 14.866 | 4 | .005 |  |  |  |
| Plaque radiother. | -.534 | .407 | 1.719 | 1 | .190 | .586 | .264 | 1.302 |
| Proton beam | -.823 | .377 | 4.757 | 1 | .029 | .439 | .209 | .920 |
| Resection | -1.221 | .415 | 8.642 | 1 | .003 | .295 | .131 | .666 |
| Other | -.285 | .476 | .359 | 1 | .549 | .752 | .296 | 1.911 |
| Chromosome 3 status |  |  | 40.676 | 2 | .000 |  |  |  |
| D3 | -1.516 | .294 | 26.544 | 1 | .000 | .219 | .123 | .391 |
| M3 | .252 | .183 | 1.891 | 1 | .169 | 1.286 | .898 | 1.841 |

**ENTRY OF 6-MONTH PREDICTORS**

| **Omnibus Tests of Model Coefficients^a^** | | | | | | | | | |
| --- | --- | --- | --- | --- | --- | --- | --- | --- | --- |
| -2 Log Likelihood | Overall (score) | | | Change From Previous Step | | | Change From Previous Block | | |
|  | Chi-square | df | Sig. | Chi-square | df | Sig. | Chi-square | df | Sig. |
| 2560.713 | 279.514 | 17 | .000 | 27.125 | 9 | .001 | 27.125 | 9 | .001 |
|  | | | | | | | | | |

| **Variables in the Equation** | | | | | | | | |
| --- | --- | --- | --- | --- | --- | --- | --- | --- |
|  | B | SE | Wald | df | Sig. | Exp(B) | 95.0% CI for Exp(B) | |
|  |  |  |  |  |  |  | Lower | Upper |
| Age | .017 | .006 | 7.201 | 1 | .007 | 1.017 | 1.005 | 1.030 |
| Tumor diameter | .187 | .022 | 70.286 | 1 | .000 | 1.205 | 1.154 | 1.259 |
| Treatment |  |  | 14.566 | 4 | .006 |  |  |  |
| Plaque radiother. | -.475 | .419 | 1.289 | 1 | .256 | .622 | .274 | 1.412 |
| Proton beam | -.798 | .387 | 4.248 | 1 | .039 | .450 | .211 | .962 |
| Resection | -1.206 | .425 | 8.060 | 1 | .005 | .299 | .130 | .688 |
| Other | -.280 | .494 | .321 | 1 | .571 | .756 | .287 | 1.990 |
| Chromosome 3 status |  |  | 37.705 | 2 | .000 |  |  |  |
| D3 | -1.480 | .295 | 25.106 | 1 | .000 | .228 | .128 | .406 |
| M3 | .246 | .189 | 1.696 | 1 | .193 | 1.279 | .883 | 1.851 |
| 6m Anxiety | -.101 | .029 | 12.484 | 1 | .000 | .904 | .855 | .956 |
| 6m Depression | .090 | .037 | 5.910 | 1 | .015 | 1.094 | 1.018 | 1.176 |
| 6m Symptoms | -.018 | .225 | .006 | 1 | .938 | .983 | .632 | 1.527 |
| 6m Function Limitations | .091 | .125 | .526 | 1 | .468 | 1.095 | .857 | 1.401 |
| 6m Worry Cancer Recur. | -.079 | .099 | .640 | 1 | .424 | .924 | .761 | 1.121 |
| 6m FACT G Physical | -.002 | .027 | .005 | 1 | .946 | .998 | .947 | 1.052 |
| 6m FACT G Social | -.028 | .014 | 3.907 | 1 | .050 | .974 | .949 | 1.000 |
| 6m FACT G Emotional | -.035 | .024 | 2.147 | 1 | .143 | .965 | .921 | 1.012 |
| 6m FACT G Functional | .007 | .018 | .131 | 1 | .717 | 1.007 | .972 | 1.043 |

**ENTRY OF 12-MONTH PREDICTORS**

| **Omnibus Tests of Model Coefficients^a^** | | | | | | | | | |
| --- | --- | --- | --- | --- | --- | --- | --- | --- | --- |
| -2 Log Likelihood | Overall (score) | | | Change From Previous Step | | | Change From Previous Block | | |
|  | Chi-square | df | Sig. | Chi-square | df | Sig. | Chi-square | df | Sig. |
| 2537.100 | 303.948 | 26 | .000 | 23.614 | 9 | .005 | 23.614 | 9 | .005 |
|  | | | | | | | | | |

| **Variables in the Equation** | | | | | | | | |
| --- | --- | --- | --- | --- | --- | --- | --- | --- |
|  | B | SE | Wald | df | Sig. | Exp(B) | 95.0% CI for Exp(B) | |
|  |  |  |  |  |  |  | Lower | Upper |
| Age | .019 | .007 | 8.138 | 1 | .004 | 1.019 | 1.006 | 1.032 |
| Tumor diameter | .192 | .023 | 71.027 | 1 | .000 | 1.211 | 1.158 | 1.267 |
| Treatment |  |  | 14.358 | 4 | .006 |  |  |  |
| Plaque radiother. | -.575 | .429 | 1.799 | 1 | .180 | .562 | .243 | 1.304 |
| Proton beam | -.905 | .395 | 5.254 | 1 | .022 | .405 | .187 | .877 |
| Resection | -1.261 | .432 | 8.517 | 1 | .004 | .283 | .122 | .661 |
| Other | -.380 | .500 | .577 | 1 | .447 | .684 | .256 | 1.823 |
| Chromosome 3 status |  |  | 38.116 | 2 | .000 |  |  |  |
| D3 | -1.508 | .297 | 25.729 | 1 | .000 | .221 | .124 | .396 |
| M3 | .236 | .195 | 1.471 | 1 | .225 | 1.266 | .865 | 1.854 |
| 6m Anxiety | -.104 | .037 | 8.016 | 1 | .005 | .901 | .838 | .968 |
| 6m Depression | .102 | .045 | 5.136 | 1 | .023 | 1.108 | 1.014 | 1.210 |
| 6m Symptoms | .048 | .270 | .032 | 1 | .858 | 1.050 | .618 | 1.783 |
| 6m Function Limitations | -.010 | .157 | .004 | 1 | .949 | .990 | .728 | 1.346 |
| 6m Worry Cancer Recur. | -.040 | .117 | .119 | 1 | .731 | .960 | .763 | 1.209 |
| 6m FACT G Physical | .044 | .030 | 2.151 | 1 | .142 | 1.045 | .985 | 1.109 |
| 6m FACT G Social | -.046 | .017 | 7.105 | 1 | .008 | .955 | .924 | .988 |
| 6m FACT G Emotional | -.021 | .026 | .687 | 1 | .407 | .979 | .930 | 1.030 |
| 6m FACT G Functional | .011 | .020 | .287 | 1 | .592 | 1.011 | .972 | 1.051 |
| 12m Anxiety | .006 | .034 | .030 | 1 | .862 | 1.006 | .941 | 1.076 |
| 12m Depression | -.075 | .043 | 3.110 | 1 | .078 | .928 | .853 | 1.008 |
| 12m Symptoms | -.235 | .294 | .636 | 1 | .425 | .791 | .444 | 1.408 |
| 12m Function Limitations | .120 | .175 | .474 | 1 | .491 | 1.128 | .800 | 1.590 |
| 12m Worry Cancer Recur. | -.154 | .141 | 1.198 | 1 | .274 | .857 | .650 | 1.130 |
| 12m FACT G Physical | -.071 | .027 | 6.770 | 1 | .009 | .932 | .883 | .983 |
| 12m FACT G Social | .033 | .018 | 3.532 | 1 | .060 | 1.034 | .999 | 1.071 |
| 12m FACT G Emotional | -.066 | .027 | 5.901 | 1 | .061 | .956 | .897 | 1.017 |
| 12m FACT G Functional | -.029 | .022 | 1.759 | 1 | .185 | .971 | .930 | 1.014 |

**ENTRY OF 24-MONTH PREDICTORS**

| **Omnibus Tests of Model Coefficients^a^** | | | | | | | | | |
| --- | --- | --- | --- | --- | --- | --- | --- | --- | --- |
| -2 Log Likelihood | Overall (score) | | | Change From Previous Step | | | Change From Previous Block | | |
|  | Chi-square | df | Sig. | Chi-square | df | Sig. | Chi-square | df | Sig. |
| 2524.138 | 320.900 | 35 | .000 | 12.962 | 9 | .164 | 12.962 | 9 | .164 |
|  | | | | | | | | | |

| **Variables in the Equation** | | | | | | | | |
| --- | --- | --- | --- | --- | --- | --- | --- | --- |
|  | B | SE | Wald | df | Sig. | Exp(B) | 95.0% CI for Exp(B) | |
|  |  |  |  |  |  |  | Lower | Upper |
| Age | .019 | .007 | 7.954 | 1 | .005 | 1.020 | 1.006 | 1.033 |
| Tumor diameter | .187 | .023 | 65.006 | 1 | .000 | 1.206 | 1.152 | 1.262 |
| Treatment |  |  | 15.099 | 4 | .005 |  |  |  |
| Plaque radiother. | -.615 | .436 | 1.992 | 1 | .158 | .541 | .230 | 1.270 |
| Proton beam | -.920 | .400 | 5.299 | 1 | .021 | .398 | .182 | .872 |
| Resection | -1.310 | .438 | 8.962 | 1 | .003 | .270 | .114 | .636 |
| Other | -.312 | .509 | .375 | 1 | .540 | .732 | .270 | 1.987 |
| Chromosome 3 status |  |  | 38.717 | 2 | .000 |  |  |  |
| D3 | -1.502 | .298 | 25.375 | 1 | .000 | .223 | .124 | .400 |
| M3 | .272 | .198 | 1.876 | 1 | .171 | 1.312 | .890 | 1.935 |
| 6m Anxiety | -.100 | .039 | 6.747 | 1 | .009 | .905 | .839 | .976 |
| 6m Depression | .107 | .048 | 4.852 | 1 | .028 | 1.112 | 1.012 | 1.223 |
| 6m Symptoms | .008 | .296 | .001 | 1 | .978 | 1.008 | .565 | 1.800 |
| 6m Function Limitations | .040 | .163 | .061 | 1 | .804 | 1.041 | .756 | 1.435 |
| 6m Worry Cancer Recur. | -.038 | .123 | .096 | 1 | .756 | .963 | .757 | 1.225 |
| 6m FACT G Physical | .072 | .033 | 4.795 | 1 | .029 | 1.074 | 1.008 | 1.146 |
| 6m FACT G Social | -.051 | .018 | 8.389 | 1 | .004 | .950 | .917 | .983 |
| 6m FACT G Emotional | -.038 | .028 | 1.885 | 1 | .170 | .963 | .912 | 1.016 |
| 6m FACT G Functional | .014 | .021 | .438 | 1 | .508 | 1.014 | .974 | 1.056 |
| 12m Anxiety | .014 | .038 | .129 | 1 | .720 | 1.014 | .941 | 1.092 |
| 12m Depression | -.083 | .046 | 3.274 | 1 | .070 | .921 | .842 | 1.007 |
| 12m Symptoms | -.225 | .320 | .495 | 1 | .482 | .799 | .427 | 1.494 |
| 12m Function Limitations | .222 | .206 | 1.154 | 1 | .283 | 1.248 | .833 | 1.870 |
| 12m Worry Cancer Recur. | -.142 | .151 | .886 | 1 | .347 | .867 | .645 | 1.167 |
| 12m FACT G Physical | -.067 | .029 | 5.444 | 1 | .020 | .935 | .884 | .989 |
| 12m FACT G Social | .039 | .019 | 4.209 | 1 | .040 | 1.040 | 1.002 | 1.080 |
| 12m FACT G Emotional | -.063 | .028 | 5.029 | 1 | .025 | .939 | .888 | .992 |
| 12m FACT G Functional | -.016 | .023 | .486 | 1 | .486 | .984 | .941 | 1.030 |
| 24m Anxiety | -.017 | .037 | .213 | 1 | .644 | .983 | .915 | 1.057 |
| 24m Depression | -.021 | .042 | .249 | 1 | .617 | .979 | .901 | 1.064 |
| 24m Symptoms | -.055 | .314 | .031 | 1 | .860 | .946 | .512 | 1.750 |
| 24m Function Limitations | -.223 | .190 | 1.374 | 1 | .241 | .801 | .552 | 1.161 |
| 24m Worry Cancer Recur. | .017 | .157 | .012 | 1 | .914 | 1.017 | .747 | 1.384 |
| 24m FACT G Physical | -.065 | .024 | 7.072 | 1 | .008 | .938 | .894 | .983 |
| 24m FACT G Social | .004 | .018 | .052 | 1 | .819 | 1.004 | .969 | 1.041 |
| 24m FACT G Emotional | .028 | .033 | .741 | 1 | .389 | 1.028 | .965 | 1.096 |
| 24m FACT G Functional | -.037 | .021 | 3.086 | 1 | .079 | .964 | .925 | 1.004 |
